# Medical treatment of second-trimester fetal miscarriage; A retrospective analysis

**DOI:** 10.1371/journal.pone.0182198

**Published:** 2017-07-28

**Authors:** Maarit Niinimäki, Maarit Mentula, Reetta Jahangiri, Jaana Männistö, Annina Haverinen, Oskari Heikinheimo

**Affiliations:** 1 Department of Obstetrics and Gynecology, PEDEGO Research Unit, Medical Research Center Oulu, University Hospital of Oulu and University of Oulu, Oulu, Finland; 2 Department of Obstetrics and Gynecology, University of Helsinki, and Helsinki University Hospital/Kätilöopisto Hospital, Helsinki, Finland; National Academy of Medical Sciences, NEPAL

## Abstract

**Objectives:**

Research on the treatment of second-trimester miscarriages is scarce. We studied the outcomes, and the factors associated with adverse events and need for hospital resources in the medical treatment of second-trimester miscarriage.

**Materials and methods:**

In these retrospective analyses we studied women treated for spontaneous fetal miscarriage with misoprostol-only (*n* = 24) or mifepristone and misoprostol (*n* = 177) in duration of gestation 12+1–21+6. Primary outcomes were the risk factors for surgical evacuation and excessive bleeding. Secondary outcomes were total misoprostol dose, time to expulsion and the length of hospital stay.

**Results:**

History of surgical evacuation of the uterus increased the risk of surgical evacuation (*p* = 0.027). Excessive bleeding was not associated with any of the studied variables. More misoprostol was needed when the duration of gestation exceeded 17+0 weeks (*p* = 0.036). In multivariate analysis the time to fetal expulsion was shorter in women with history of 1–2 deliveries (hazard ratio [HR] 1.49, 95% confidence interval [CI]; 1.07–2.07), ≥3 deliveries (HR 1.63, 95% CI; 1.11–2.38) and with a two-day interval between mifepristone-misoprostol administration (HR 1.71, 95% CI; 1.05–2.81). Patients with symptoms (i.e. uterine bleeding or pain) at baseline had longer hospital stay (HR 0.66, 95% CI; 0.47–0.92).

**Conclusions:**

The factors affecting the outcomes of medical treatment of second-trimester fetal miscarriage are similar to those of second-trimester induced abortion. Two-day interval between mifepristone-misoprostol administration might decrease the time to fetal expulsion and the need of hospital resources.

## Introduction

Miscarriage is a common event in a woman’s life. Approximately 20% of clinically recognized pregnancies end in a miscarriage [1]. Most miscarriages occur before 12 weeks and it has been estimated that only 2–3% of pregnancies end spontaneously in the second trimester [2]. Second-trimester pregnancy loss has been defined as miscarriage diagnosed between the duration of gestation of 12–24 weeks [2]. The Special Interest Group for early pregnancy in European Society of Human Reproduction and Embryology (ESHRE) has made a consensus statement to harmonize inconsistent terminology of pregnancy failure. According to the ESHRE statement the term fetal miscarriage should be used in pregnancy loss ≥ 10 weeks of gestation with a fetus measuring ≥ 33 mm on ultrasound [3]. According to the World Health Organization, miscarriage is defined as the premature loss of a fetus at < 22+0 weeks of the pregnancy and weighing < 500 g [4].

Medical management of miscarriage and induced abortion has become the gold standard in clinical practice in several northern European countries. However, the treatment of second trimester miscarriage has been addressed in only few research studies. In a recent systematic review of medical treatments for incomplete miscarriage, no randomized trials focused specifically on the treatment of miscarriage beyond 13 week´s gestation were identified [5]. Thus the clinical practice on the treatment of late miscarriages is mainly based on the knowledge derived from studies on second trimester induced abortion. Still, miscarriage can be considered a pathophysiologically separate entity compared to induced abortion, in which potentially viable fetus is expulsed with the use of medication. Women with miscarriage are a heterogeneous group, presenting with widely varying etiologies, such as infections, chromosomal abnormalities of the mother and/or the fetus, antiphospholipid syndrome and congenital uterine anomalies [2].

In a randomized study comparing the use of misoprostol and oxytocin in cases of both fetal miscarriages and induced abortions in the second trimester women with miscarriage had a shorter time to expulsion compared to women with induced abortion [6]. In randomized trials [7–9] the use of the mifepristone-misoprostol combination decreased the time to fetal expulsion and increased the rate of successful uterine evacuation, compared to use of misoprostol alone, in women with second trimester induced abortion [7–9]. In Finnish register-based study the rate of surgical evacuation was 39% after second trimester medical induced abortion including both mifepristone and misoprostol [10]. Moreover, in a randomized study comparing the effects of one- and two-day dosing intervals between mifepristone and misoprostol the rate of surgical evacuation was 31%. This risk was increased by a history of surgical evacuation, fetal indications of induced abortion, age > 24 years and a two-day dosing interval between mifepristone and misoprostol [11]. In contrast, in previous studies on medical induced abortion shorter time to fetal expulsion has been associated with parity and lower gestational age [6, 10].

We evaluated the outcomes of spontaneous second trimester miscarriages treated with misoprostol-only or with a combination of mifepristone and misoprostol. The primary outcome was to identify the factors associated with adverse outcomes (the need of surgical evacuation and excessive bleeding). The secondary outcome was to investigate the need of hospital resources (misoprostol dose, time to fetal expulsion and length of hospital stay).

## Materials and methods

The study was conducted between 1^st^ December 2008 and 31^st^ December 2011 in two Finnish tertiary care hospitals; the Departments of Obstetrics and Gynecology at the Helsinki and Oulu University Hospitals. The setting was retrospective. The data was collected by three authors (MM, RJ, AH) and fully deindetified before accessed and analysed by MN. A systematic search was conducted in both hospitals wherein data were collected on all patients treated with ICD10-codes O02.X or O03.X. Of these women, those with a spontaneous fetal miscarriage (a non-viable fetus visible in the ultrasound examination) and duration of pregnancy between 12+1–21+6 gestational weeks were included. Multiple pregnancies were excluded. In cases with several miscarriages per woman during the study period, only the first miscarriage was included. Three patients were excluded as they were treated with intravenous oxytocin only. One patient was excluded because of missing information on the treatment at the ward (number of misoprostol doses and time to fetal expulsion). Three patients experienced fetal expulsion following mifepristone-only and were included in the mifepristone-misoprostol group. Using these criteria we identified altogether 150 women in the Helsinki University Hospital and 51 women in the Oulu University Hospital.

We collected data from individual patient files on the age of the woman at the time of diagnosis, previous pregnancies, vaginal deliveries, miscarriages, induced abortions and surgical evacuations. The symptoms (abdominal pain, cramps and bleeding) experienced at the time of diagnosis were also collected. According to Finnish practice, all fetuses are scanned using ultrasonography to confirm the miscarriage. However, the duration of pregnancy was calculated from the last menstrual period as we found it clinically more relevant than ultrasound definition of the fetus although some discrepancy may occur. We assumed that in cases of intrauterine death the size of the fetus may not the match the duration of gestation due to fetal growth restriction or fetal decay, and thus it is not any more accurate method. Data were also collected on the treatment, specifically on mifepristone administration and the interval between the administration of the mifepristone dose and hospital admittance, total dose and number of misoprostol doses, volume of bleeding (as estimated by the staff on the ward and in the operation room) and time to fetal expulsion (calculated from the first misoprostol dose) and length of the hospital stay (overnight treatment from the day when the woman attended the ward to receive misoprostol). Bleeding exceeding 1000 ml was considered excessive.

Most patients (*n* = 177) received 200 mg of mifepristone orally at the first outpatient visit. Thereafter, they were admitted to the ward 0–72 hours later and the first misoprostol dose was administered vaginally. The initial dose of misoprostol was 400–800 μg. Repeat vaginal doses (400 μg) were given at three-hour intervals until fetal expulsion was achieved. However, 24 patients received repeated doses of misoprostol-only without pretreatment with mifepristone.

The Department of Obstetrics and Gynecology, and Pediatrics of the Hospital District of Helsinki and Uusimaa granted a permission for the study (§17/24.05.2011). According to Finnish law, a medical research ethics committee statement is not required in a clinical study where the clinical information has been obtained through a retrospective audit and the patients have not been contacted for the purposes of performing the study.

### Statistical analyses

Statistical analyses were performed using IBM SPSS^®^ Statistics 22 software. The chi-square test was used for a comparison of the categorical variables. The Mann-Whitney U-test or Kruskal-Wallis tests were employed for analysis of the continuous variables. Cox´s regression was performed to evaluate the time to successful events, i.e. misoprostol dose, time to fetal expulsion and length of hospital stay. Univariate analyses were performed initially. Data indicating statistical significance were further evaluated using multivariate analysis. Multicollinearity was tested by using a linear regression test. A variance inflation factor (VIF) >2.5 was interpreted as multicollinearity between the variables. Accordingly, the relevant data were discarded from the multivariate analysis. The level of statistical significance was set at *P* < 0.05.

## Results

201 women with second trimester miscarriage met the inclusion criteria. Their mean age (± standard deviation [SD]) was 32.4 (±5.5) years. The current pregnancy was the first for 34 women (17%), 109 women had had 2–4 previous pregnancies (54%) and 58 women had had ≥ 5 previous pregnancies (29%). Of these women 177 (88%) were treated with the combination of mifepristone and misoprostol. The remaining women (*n* = 24) were treated with misoprostol-only. Mean duration of gestation was 117.1 days when defined by the last menstrual period and 103.4 days according to the size of the fetus in the ultrasonographic examination at the time of diagnosis. The results did not alter significantly, when calculated according to the ultrasonographic dating of gestation.

The primary outcomes (surgical evacuation during the first hospital stay and bleeding exceeding 1000 ml) were analysed according to the background characteristics and the use of mifepristone (Table 1). Surgical evacuation of the uterus because of bleeding and/or retained products of conception was performed during the first hospital stay in 51 patients (25%) and within eight weeks in 65 (32%). In all cases fetus had been expulsed before surgical evacuation. Bleeding was estimated to be excessive in 54 women (27%). A history of previous surgical evacuation of the uterus (*p* = 0.027) was associated with the need for surgical evacuation. None of the parameters studied were associated with excessive bleeding.

**Table 1:** Patient characteristics, use of mifepristone and the primary outcomes.

	Surgical evacuation		Exessive bleeding (estimated > 1000 ml)	
	(n,%)	p	(n,%)	p
Age, years (n)				
less than 25 (17)	1 (5.9)	0.186	1 (5.9)	0.322
25–29 (38)	9 (23.7)		10 (26.3)	
30–34 (70)	16 (22.9)		19 (27.1)	
35–39 (57)	18 (31.6)		18 (31.6)	
40 or more (19)	7 (36.8)		6 (31.6)	
Previous pregnancies (n)				
1 (34)	7 (20.6)	0.080	7 (20.6)	0.153
2 to 4 (109)	23 (21.1)		26 (23.9)	
5 or more (58)	21 (36.2)		21 (36.2)	
Previous vaginal deliveries* (n)				
0 (64)	14 (21.9)	0.701	14 (21.9)	0.423
1 to 2 (86)	24 (27.9)		27 (31.4)	
3 or more (50)	13 (26.0)		13 (26.0)	
Previous miscarriages (n)				
0 (131)	33 (25.2)	0.502	35 (26.7)	0.593
1 to 2 (57)	13 (22.8)		14 (24.6)	
3 or more (13)	5 (38.5)		5 (38.5)	
Previous induced abortions (n)				
No (177)	43 (24.3)	0.340	46 (26.0)	0.446
Yes (24)	8 (33.3)		8 (33.3)	
Previous surgical evacuations of the uterus (n)				
No (163)	36 (22.1)	0.027	41 (25.2)	0.257
Yes (38)	15 (39.5)		13 (34.2)	
Duration of gestation, weeks (n)				
12+1–16+6 (112)	29 (25.9)	0.849	29 (25.9)	0.727
17+0–21+6 (89)	22 (24.7)		25 (28.1)	
Symptoms at the baseline (n)				
No (141)	37 (26.2)	0.665	37 (26.2)	0.759
Yes (60)	14 (23.3)		17 (28.3)	
Mifepristone administration				
No (24)	6 (25.0)	0.964	8 (33.3)	0.446
Yes (177)	45 (25.4)		46 (26.0)	
Mifepristone-misoprostol interval, days (n)				
0 (25)	5 (20.0)	0.799	7 (28.0)	0.560
1(116)	30 (25.9)		28 (24.1)	
2 (60)	16 (26.7)		19 (31.7)	

* 1 missing value

We analysed the need for hospital resources according to the different parameters; total misoprostol dose, time to fetal expulsion and length of hospital stay (Table 2). The total dose of misoprostol needed was significantly higher when the duration of gestation was ≥ 17+0 weeks (*p* = 0.036). Time to expulsion was shorter in women with a history of pregnancy (*p* = 0.010) and delivery (*p* = 0.004) with a greater interval between the administration of mifepristone and misoprostol (*p* = 0.016) and when the duration of pregnancy was < 17+0 weeks (*p* = 0.023). Length of hospital stay was shorter in women with a history of delivery (*p* = 0.010), in women who received mifepristone (*p* = 0.003) and in those in for whom the dosing interval between the administration of mifepristone and misoprostol was greater (*p* = 0.003). Women with symptoms at baseline experienced a longer hospital stay than asymptomatic women (*p* < 0.001). A longer duration of gestation was associated with a longer hospital stay (*p* = 0.006).

**Table 2:** Need for hospital resources (misoprostol dose, time to expulsion and the length of hospital stay).

	Misoprostol dose mg, mean (median, SD)	p	Time to expulsion* (minutes), mean (median, SD)	p	Length of hospital stay (days), mean (median, SD)	p
Age, years (N)						
less than 25 (17)	1.29 (1.20, 0.63)	0.861	489.4 (360.0, 373.5)	0.422	1.53 (1.00, 0.62)	0.614
25–29 (38)	1.13 (1.20, 0.39)		398.6 (357.5, 200.2)		1.55 (1.00, 1.22)	
30–34 (70)	1.11 (1.20, 0.40)		390.0 (350.0, 216.7)		1.79 (1.00, 1.22)	
35–39 (57)	1.11 (1.20, 0.38)		363.4 (320.0, 199.7)		1.79 (2.00, 1.16)	
40 or more (19)	1.27 (0.80, 1.03)		330.3 (330.0, 252.9)		1.79 (1.00, 1.40)	
Previous pregnancies (N)						
1 (34)	1.22 (1.20, 0.46)	0.110	475.1 (440.0, 256.3)	0.010	1.97 (2.00, 1.19)	0.076
2 to 4 (109)	1.12 (0.80, 0.56)		381.4 (330.0, 228.0)		1.69 (1.00, 1.30)	
5 or more (58)	1.15 (1.20, 0.39)		345.0 (310.0, 206.1)		1.64 (1.50, 0.87)	
Previous vaginal deliveries* (N)						
0 (64)	1.22 (1.20, 0.63)	0.198	444.6 (402.5, 239.4)	0.004	2.00 (2.00, 1.30)	0.010
1 to 2 (86)	1.08 (0.80, 0.43)		374.0 (330.0, 227.0)		1.61 (1.00, 1.28)	
3 or more (50)	1.16 (1.20, 0.40)		335.2 (270.0, 213.2)		1.58 (1.00, 0.70)	
Symptoms at the baseline (N)						
No (141)	1.10 (1.20, 0.35)	0.445	370.4 (330.0, 193.6)	0.313	1.47 (1.00, 0.72)	<0.001
Yes (60)	1.25 (1.20, 0.75)		430.0 (345.0, 301.5)		2.32 (2.00, 1.71)	
Mifepristone administration						
No (24)	1.23 (1.20, 0.67)	0.663	434.5 (375.0, 339.0)	0.602	2.46 (2.00, 1.64)	0.003
Yes (177)	1.13 (1.20, 0.47)		380.9 (330.0, 211.4)		1.62 (1.00, 1.07)	
Mifepristone-misoprostol interval, days (N)						
0 (25)	1.25 (1.20, 0.66)	0.281	431.8 (362.5, 340.0)	0.016	2.40 (2.00, 1.61)	0.003
1(116)	1.17 (1.20, 0.51)		410.7 (357.5, 214.2)		1.74 (1.00, 1.23)	
2 (60)	1.05 (0.80, 0.37)		324.4 (290.0, 194.9)		1.40 (1.00, 0.62)	
Duration of gestation, weeks (N)						
12+1–16+6 (113)	1.09 (1.00, 0.50)	0.036	357.4 (320.0, 211.2)	0.023	1.55 (1.00, 1.02)	0.006
17+0–21+6 (92)	1.21 (1.20, 0.49)		423.9 (370.0, 247.8)		1.94 (2.00, 1.32)	

SD = standard deviation

Mg = milligrams

*Time from the first misoprostol dose to expulsion of the fetus

The association between mifepristone administration, and the absence or presence of symptoms at the time of diagnosis was also analyzed. Mifepristone was used less frequently in symptomatic than in asymptomatic women (71.9% vs. 92.9%, *p* = 0.001). In addition, the mifepristone-misoprostol interval was shorter in symptomatic women. The dosing interval was 0, 1 or 2 days in 11 (7.8%), 80 (56.7%) and 50 (35.3%) asymptomatic women and 14 (23.3%), 36 (60.0%) and 10 (16.7%) symptomatic women, respectively (*p* = 0.001).

Cox´s regression analysis was carried out to examine, which parameters were associated with the total misoprostol dose, time to fetal expulsion and the length of hospital stay (Table 3). None of the variables had a significant association with the misoprostol dose in univariate analysis. In contrast, univariate analysis revealed a significant association between the time to expulsion and the number of previous pregnancies and vaginal deliveries, the mifepristone-misoprostol interval and the duration of gestation. Number of previous pregnancies and deliveries showed multicollinearity. Thus, we chose to discard the number of previous pregnancies from the multivariate analysis. Of the three parameters included in the multivariate model, a history of 1–2 (hazard ratio [HR] 1.49, 95% confidence interval [CI];1.07–2.07) or ≥ 3 vaginal deliveries (HR 1.63, 95% CI; 1.11–2.38) and a two-day dosing interval between mifepristone and misoprostol (HR 1.71, 95% CI; 1.05–2.81) were associated with a shorter time to expulsion. In univariate analysis symptoms and mifepristone interval were associated with the length of hospital stay, and were entered to multivariate model, in which women with symptoms had longer duration of hospital stay (HR 0.66, 95% CI; 0.47–0.92).

**Table 3:** Cox´s regression of time to expulsion and length of hospital stay (univariate and multivariate analysis).

	Univariate analysis		Multivariate analysis	
	HR (95% CI)	p	HR (95% CI)	p
**Time to expulsion (minutes)**				
Previous pregnancies (N) *				
1 (34)	1			
2 to 4 (109)	1.43 (0.97–2.11)	0.069		
5 or more (58)	1.68 (1.09–2.51)	0.018		
Previous vaginal deliveries (N)				
0 (65)	1		1	
1 or 2 (89)	1.38(0.99–1.92)	0.053	1.49 (1.07–2.07)	0.019
3 or more (50)	1.63 (1.12–2.38)	0.011	1.63 (1.11–2.38)	0.012
Mifepristone-misoprostol interval, days (N)				
0 (25)	1		1	
1 (116)	1.15 (0.73–1.81)	0.542	1.19 (0.76–1.88)	0.446
2 (60)	1.66 (1.02–2.70)	0.041	1.71 (1.05–2.81)	0.033
Duration of gestation, weeks (N)				
12+1–16+6 (113)	1		1	
17+0–21+6 (92)	0.75 (0.56–0.99)	0.044	0.76 (0.57–1.01)	0.057
**Length of hospital stay (days)**				
Symptoms at the baseline (N)				
No (141)	1		1	
Yes (60)	0.62 (0.45–0.85)	0.003	0.66 (0.47–0.92)	0.015
Mifepristone-misoprostol interval, days (N)				
0 (25)	1		1	
1(116)	1.35 (0.87–2.09)	0.176	1.22 (0.79–1.91)	0.371
2 (60)	1.68(1.04–2.70)	0.034	1.41 (0.86–2.31)	0.178

*Discarded from multivariate analysis due to multicollinearity with vaginal deliveries

1 = reference group

HR = Hazard Ratio

CI = Confidence Interval

## Discussion

History of surgical evacuation of the uterus was the only parameter associated with the risk of further surgical evacuation following medical treatment of fetal miscarriage. There might be some underlying explanations, such as insufficient maturing of the cervix or lower uterine contractility, which unfortunately cannot be further studied in the present study setting.

In contrast, no risk factors for excessive bleeding could be identified. When the duration of gestation exceeded 17+0 weeks, a higher total dose of misoprostol was needed. Time to fetal expulsion was shorter in parous women and when the mifepristone-misoprostol interval was two days, compared to a zero-day interval. The length of hospital stay was longer in symptomatic women than in asymptomatic women. We speculate that this is due to the cautious pretreatment of symptomatic women with mifepristone. The symptoms experienced could be interpreted as signs of spontaneous onset of miscarriage and led to the non-use of mifepristone or to the use of a short mifepristone-misoprostol interval. In fact, symptomatic women received mifepristone less frequently, and the dosing interval between mifepristone and misoprostol administration was shorter. Thus, these women might also benefit from pretreatment with mifepristone.

As the existing data on the treatment of second-trimester miscarriages are scarce, and national or international guidelines are lacking, we found the present analysis important. In order to collect sufficient data, the patents were collected from two large Finnish tertiary care hospitals. The retrospective nature of the study is a shortcoming. Second-trimester miscarriages are a rare, and prospective studies would need to be conducted over a greater length of time and involve high patient volumes.

Given the rarity of late miscarriage, the different treatment protocols for second trimester fetal miscarriage have not been assessed in prospective trials. A systematic review and meta-analyses aiming to establish recommendation for post-abortion care in second trimester showed that pretreatment with mifepristone was associated with shorter expulsion of the fetus. However, the articles included were heterogeneous including both induced abortions (legal or illegal) and spontaneous pregnancy failures (fetal demise, ruptured membranes and incomplete abortion) [12]. The treatment protocol used for spontaneous fetal miscarriages in Finland is in line with recent WHO recommendations for medical termination of pregnancy after 12 weeks of gestation [13]. However, in the absence of randomized clinical trials or clinical guidelines not all patients receive similar care. For example, in the presence of clinical symptoms suggestive of spontaneous abortion the clinician may decide not to administer mifepristone prior to misoprostol. This was seen in 12% of cases in the present study.

Generally, factors affecting the outcome of second-trimester spontaneous fetal miscarriage treated medically were found to be similar to those in second-trimester medical induced abortion. In a prospective randomized trial the history of surgical evacuation of the uterus was also identified as a risk factor for surgical evacuation in second-trimester medical induced abortion [11]. Nulliparous women were likely to experience a longer induction-to-abortion interval than uni- or multiparous women in second-trimester medical induced abortion in another retrospective study [14]. Unlike the findings of the study on medical induced abortion by Abbas *et al*. (2016), the mifepristone-misoprostol interval was not associated with the misoprostol dose in our study [15]. However, the time to fetal expulsion was shorter in relation to increased mifepristone-misoprostol interval and also in women with a history of vaginal delivery. This has also been observed in medical second-trimester induced abortion [15]. The two-day interval between mifepristone and misoprostol has been shown to associate with shorter time to expulsion and also a lower incidence of surgical evacuation, compared to one-day interval in second-trimester medical induced abortion [16]. According to other studies, administration interval of 36–48 hours interval was considered preferable in second-trimester induced abortion [17].

Several studies have provided convincing evidence on the advantages of combined mifepristone-misoprostol protocol, instead misoprostol-only in second-trimester induced abortion [7–9,18]. In cases of spontaneous fetal miscarriage, the practice is not clear. It seems that the results of studies on second-trimester induced abortion can also be generalized to fetal miscarriages. As guidelines are lacking, treatment protocols used in second trimester medical induced abortion can also be applied to second-trimester missed miscarriage. Based on our results, the use of mifepristone, and especially a longer mifepristone-misoprostol interval might reduce the time to fetal expulsion. Multicenter studies using a prospective randomized setting are needed to confirm these findings.

## References

[1] NICE Clinical guideline 2012. Diagnosis and initial management in early pregnancy of ectopic pregnancy and miscarriage. 23638497

[2] McNamee KM, Dawood F, Farquharson RG. Mid-trimester pregnancy loss. Obstet Gynecol Clinics North America 2014;41(1):87–102. 10.1016/j.ogc.2013.10.007 24491985

[3] Kolte AM, Bernardi LA, Christiansen OB, Quenby S, Farquharson RG, Goddijn M, et al Terminology for pregnancy loss prior to viability: a consensus statement from the ESHRE early pregnancy special interest group. Hum Reprod 2015;30(3):495–8. doi: 10.1093/humrep/deu299 25376455 10.1093/humrep/deu299

[4] WHO Regional Strategy on Sexual and Reproductive Health. Definitions and indicators in family planning, maternal and child health and reproductive health. Geneva: World Health Organization, 2001.

[5] Kim C, Barnard S, Neilson JP, Hickey M, Vazquez JC, Dou L. Medical treatment for incomplete miscarriage. Cochrane Database of Systematic Reviews 2017, Issue 1.Art. No.:CD007223. 10.1002/14651858.CD007223.pub4 PMC6464743 28138973

[6] Elami-Suzin M, Freeman MD, Porat N, Rojansky N, Laufer N, Ben-Meir A. Mifepristone followed by misoprostol or oxytocin for second-trimester abortion. Obstet Gynecol 2013;122(4):815–20. doi: 10.1097/AOG.0b013e3182a2dcb7 24084539 10.1097/AOG.0b013e3182a2dcb7

[7] Ngoc NTN, Shochet T, Raghavan S, Blum J, Nga NTB, Minh NTH, et al Mifepristone and misoprostol compared with misoprostol alone for second-trimester abortion. Obstet Gynecol 2011;118(3):601–8. doi: 10.1097/AOG.0b013e318227214e 21860289 10.1097/AOG.0b013e318227214e

[8] Dabash R, Chelli H, Hajri S, Shochet T, Raghavan S, Winikoff B. A double-blind randomized controlled trial of mifepristone or placebo before buccal misoprostol for abortion at 14–21 weeks of pregnancy. Int J Gynecol Obstet 2015;130:40–4. 10.1016/j.ijgo.2015.02.023 25896965

[9] Kapp N, Borgatta L, Stubblefield P, Vragovic O, Moreno N. Mifepristone in second-trimester medical abortion: a randomized controlled trial. Obstet Gynecol 2007;110(6):1304–10. doi: 10.1097/01.AOG.0000289577.32274.a5 18055725 10.1097/01.AOG.0000289577.32274.a5

[10] Mentula M, Suhonen S, Heikinheimo O. One- and two-day dosing intervals between mifepristone and misoprostol in second trimester medical termination of pregnancy–a randomized trial. Hum Reprod 2011;26(10):2690–7. doi: 10.1093/humrep/der218 21798991 10.1093/humrep/der218

[11] Mentula M, Heikinheimo O. Risk factors of surgical evacuation following second-trimester medical termination of pregnancy. Contraception 2012; 86(2):141–6. doi: 10.1016/j.contraception.2011.11.070 22240172 10.1016/j.contraception.2011.11.070

[12] Mark A, Edelman A, Borgatta L. Second-trimester postabortion care for ruptured membranes, fetal demise, and incomplete abortion. Int J Gynecol Obstet 2015;129:98–103. 10.1016/j.ijgo.2014.11.011 25660084

[13] Tang J, Kapp N, Dragoman M, de Souza JP. WHO recommendations for misoprostol use for obstetric and gynecologic indications. Int J Gynecol Obstet 2013;121:186–9. 10.1016/j.ijgo.2012.12.009 23433680

[14] Joensuu-Manninen H, Kuvaja P, Talvensaari-Mattila A. Clinical efficacy of mifepristone and misoprostol in second-trimester pregnancy termination. Acta Obstet Gynecol 2010;89:1552–6. 10.3109/00016349.2010.513427 21080899

[15] Abbas DF, Blum J, Ngoc NT, Nga NT, Chi HT, Martin R, et al Simultaneous administration compared with a 24-hour mifepristone-misoprostol interval in second-trimester abortion: a randomized controlled trial. Obstet Gynecol 2016;128(5):1077–83. doi: 10.1097/AOG.0000000000001688 27741182 10.1097/AOG.0000000000001688

[16] Heikinheimo O, Suhonen S, Haukkamaa M. One- and 2-day mifepristone-misoprostol intervals are both effective in medical termination of second-trimester pregnancy. Reprod Biomed Online 2004;8(2):236–9. 14989807 10.1016/s1472-6483(10)60522-6

[17] Gemzell-Danielsson K, Lalitkumar S. Second-trimester medical abortion with mifepristone-misoprostol and misoprostol alone: a review of methods and management. Reprod Health Matters 2008;16(31s):162–72. 10.1016/S0968-8080(08)31371-8 18772097

[18] Wildschut H, Both MI, Medema S, Thomee E, Wildhagen MF, Kapp N. Medical methods for mid-trimester termination of pregnancy. Cochrane Database of Systematic Reviews 2011, Issue 1. Art. No.:CD005216. 10.1002/14651858.CD005216.pub2 PMC8557267 21249669

